# Relationship among Sex, Skin Color, and Production Parameters of Broiler in Pectoral Myopathies

**DOI:** 10.3390/ani12131617

**Published:** 2022-06-23

**Authors:** Martín Novoa, Iván Francisco, Almudena Lage, José Luis Benedito, Lucio García, Luis Vázquez, Noemi Cobas

**Affiliations:** 1 COREN, Sociedad Cooperativa Galega, 32003 Orense, Spain; martin.novoa@coren.es (M.N.); ivanfrancisco.vazquez@coren.es (I.F.); almudena.lage@coren.es (A.L.); 2 Departamento de Patología Animal, Facultad de Veterinaria, Universidad de Santiago de Compostela (USC), 27002 Lugo, Spain; joseluis.benedito@usc.es; 3 Fundación Centro Tecnolóxico da Carne de Galicia, Rúa Galicia 4, Parque Tecnológico de Galicia, San Cibrao das Viñas, 32900 Ourense, Spain; luciogarcia@ceteca.net (L.G.); luisvazquez@ceteca.net (L.V.)

**Keywords:** poultry, sex, particle size, wooden breast, spaghetti meat

## Abstract

**Simple Summary:**

Genetic selection in poultry production aims to obtain more efficient and fast-growing broilers. Breast is an economically interesting poultry cut; thus, its increased development, known as hypertrophy, is a selection criterion that has been adopted by breeders. However, genetic drift is increasing breast myopathies, alterations in the normal arrangement of the muscle fibers that compose the muscle, among broilers. The altered breast meat is fit for human consumption, and it does not represent a public health issue; nevertheless, it causes important commercial losses. Among all of the possible factors that may contribute to breast myopathies, the relationship between sex, skin color, live weight, conversion rate, and feed particle size and two types of alterations (wooden breast and spaghetti meat) was evaluated. It was found that such factors significantly affected the appearance of these myopathies in most cases, influencing muscle alterations in different and even opposite ways. Although the incidence of myopathies in the breast was low in the broilers studied, it is necessary to adapt the production conditions to offer a product of the highest quality.

**Abstract:**

Breast anomalies in broilers, especially wooden breast (WB) and spaghetti meat (SM), cause high economic losses to the poultry meat sector. In order to identify the parameters that have a causal effect and to reduce the incidence of these myopathies, 141,792 broilers were analyzed in a total of 1477 batches using a visual grading system. The relationship among productive parameters such as the feed conversion ratio, live weight, growth rate, and mortality, was evaluated. Effects due to skin color (white vs. yellow), broiler sex (male, female, and mixed groups), feed presentation (grain vs. mash), and veterinary treatments (treated vs. untreated) were also included in the statistical study. Live weight was observed to have a significant effect (*p* < 0.001) on WB incidence, which increased by 1.11 for each 100 g of weight. Weight did not significantly affect the incidence of SM. Males had a higher incidence of WB and a lower incidence of SM than females. The incidence of both myopathies varied between samples that turned out to be significantly affected by some of the variables considered in the model, such as grain feeding and the feed conversion ratio. Controlling these factors in the broiler production could help to reduce the incidence of WB and SM.

## 1. Introduction

Spain is one of the top poultry-meat-producing countries in the European Union [1]. This meat can be sold as a whole carcass, cut up or processed, or ready-to-eat products. Purchase preferences have markedly changed in recent years, forcing the global broiler industry to introduce genetic selection in order to achieve the greater performance of different pieces, such as breast [2]. This tendency is largely due to consumer interest in its nutritional composition—low in fat, sodium, and cholesterol [3], its good organoleptic properties, and its suitability for easy, fast, diverse home-cooking methods [4]. Intensive production systems, fast-growing and high-breast-yield hybrids or breeds, changes in broiler diets, and genetic selection are required to achieve a high breast yield in response to this market demand [4,5,6,7,8]. For example, Schmidt et al. [9] reported an increase of 9% when comparing breast muscle yield of 5 week old chickens in a modern strain (Ross 708 broiler) with a heritage line from the University of Illinois that had not been selected since the 1950s. However, this profound change in the poultry industry has led to counterproductive consequences with respect to the hypertrophy in the pectoralis major muscle, increasing the incidence of myopathies in broiler breast meat and devaluing its quality [10,11].

The most common muscle abnormalities in breast meat are wooden breast (WB) and spaghetti meat (SM). The first one causes significant economic losses because of its low aesthetic acceptability [12], which contributes to consumer rejection [7,13]. Recent studies have pointed out its increased occurrence in chickens raised in different parts of the world, such as the USA [14], Italy [12], and the United Kingdom [15]. Depending on the affection level, the characteristics of WB include significant hardness, a paler color, hemorrhaging, and an exudate muscle surface [16]. On the other hand, SM is characterized as loss of integrity in the muscle fibers and a tendency for the fibers to separate, mainly in the cranial region of pectoral muscle [17,18]. SM results in tender, fragile, or even mushy meat [19]. The proximate composition of meat is also affected by this muscle abnormality. For example, Baldi et al. [18] reported a lower total protein content and higher moisture when compared to normal breast samples. In any case, both defects—WB and SM—translate into a reduction in technical properties [20], poor-quality meat, and low consumer acceptability. Consequently, the poultry industry is forced to downgrade this meat and use it for the production of processed foods in which the breast is chopped, or the meat is ground. This also results in corresponding economic losses. Accordingly, it is necessary to deepen our knowledge of all of the parameters implicated in the occurrence of these abnormalities in order to adopt a long-term strategy to reduce their presence and their degree.

Broiler breeding systems affect the quality of the final product [21]. For example, Kuttappan et al. [22] and Trocino et al. [7] reported that the feeding regime (ad libitum vs. restricted) could modify the occurrence of myopathies, with chicks fed ad libitum showing a lower incidence of myopathies because of a more constant growth rate during the trial. Some studies have also highlighted that high growth rates and high breast yield play an important role in muscle hypertrophy [13,23,24]. Sobotik et al. [25] described an increase in live bird weights and breast yield without an increase in WB when broiler feed was supplemented with phosphatidic acid. Córdova-Noboa et al. [26] reported that using guanidinoacetic acid improved broiler breast yield with a low manifestation of myopathies. The physical form of the feed—the nutrient intake condition and feed conversion of reared poultry [27]—could also affect the incidence of myopathies. Higher live weight and lower feed conversion rates were obtained when the poultry feed was in the form grain rather than in the form of a mash [28]. In addition, birds prefer to eat food with a small seed size; thus, using granular feed caused an increase in the daily intake [29]. However, Amerah et al. [27] indicated that broilers fed with mash had greater gizzard development and smaller intestines, increasing their digestive efficiency.

Hence, the priority for the poultry sector is to evaluate all of the parameters that are implicated in myodegeneration and to determine the most appropriate management and breeding techniques. In this regard, several authors have studied the effects of slaughtering age and other feeding parameters, such as the use of low-energy diets, feed additives, or feed restriction [7,22,26]. Nevertheless, there are not enough data on the effects of other production aspects, especially SM incidence. Thus, the aim of the present work was to evaluate the relationship between production systems and breast meat quality and analyze the occurrence of WB and SM in relation to production system parameters, such as breed, farm management, animal density, and food particle size.

## 2. Materials and Methods

### 2.1. Data Collection

Data were collected from January 2016 to September 2017. This study was carried out on broilers from a total of 213 farms running six production cycles per year. All of the slaughterhouses were located in Ourense (Spain) and belonged to the same agri-food company (Cooperativas Orensanas SCG—Coren).

Some aspects related to broiler management mainly depend on sex (male, female, or mixed groups). Broilers of the same sex were raised with the same feed and under similar ventilation parameters and light–dark cycles. On the other hand, the color of broiler skin depends on diet, and all of the samples in the same color group followed the same feeding program. All of the farms included in this study run an intensive system with the same feeding program depending on the stages of growth, sex, and skin color. Welfare animal practices were in accordance with company recommendations, while poultry health was also regulated by the company. The slaughter methods were the same in all samples.

For transport to the slaughterhouse, the birds were always loaded and unloaded manually, and the density of birds per cage was always the same. Each batch of transport to the slaughterhouse constituted a unit of sampling.

Originally, the data file included 1540 broiler batches. However, they were filtered to remove the variables without enough data for an adequate statistical analysis, such as batches of animals with a different production system (Label). Therefore, the birds included in this study corresponded to 1477 sampling units. The animals used as experimental material came from two different broiler lines, the Ross 308 (https://es.aviagen.com/brands/ross/products/ross-308) (accessed on 3 February 2022) pure line and Cobb 500 pure line (https://www.cobb-vantress.com/es_MX/products/cobb500/) (accessed on 3 February 2022), neither of which had undergone hybridization. A total of 96 carcasses per sampling unit were randomly selected for assessment by a visual inspection.

The experiment was performed in accordance with the European Regulations for the protection of animals at the time of slaughter (Council Regulation (EC) No 1099/2009 of 24 September 2009).

### 2.2. Parameters Used in the Statistical Model

Broiler carcass conditioning included weighting the individual animals, electrical water bath stunning, neck cutting and bleeding, scalding, and plucking. Analyses included a macroscopic examination of the breasts (*pectoralis major*). Mortality (expressed as a percentage), the feed conversion ratio (FCR) (expressed as kg feed/kg live weight), and growth rate (GR) (g/day) were also determined. The following formulas were used for these parameters:

Mortality = (total dead animals per batch/total animal per batch) × 100.

FCR = Total of feed consumption per batch in kg/final alive weight of the batch.

GR = Final average alive weight per batch/growing days.

Density = Final average alive weight per farm in kg × bird per farm/surface in m^2^.

The dependent variables correspond to the incidence of two myopathies in broiler breasts—WB and SM—which are linked to the production system. These defects were evaluated with a visual inspection. According to Soglia et al. [30], the breasts were considered to be affected by WB when out-bulging, pale areas with a hardened consistency, superficial petechiae, and exudate were present in the muscle. On the other hand, the muscle was considered to be affected by SP when the fibers showed a tendency toward separation. The incidence of these myopathies was expressed as a percentage, and the coefficient of variation (CV) was used to compare the average batch results. In order to predict these variables, sex (male: M, female: F, and mixed groups: X) and skin color (white: H, and yellow: Y) were considered as fixed effects. In the special case of females, two more groups were necessary to highlight: white females in over-density (HF-O) and yellow females fed a minimum of 65% cereals (YF-65). The influence of the particle size of the feed on the incidence of pectoralis myopathies was analyzed using two factorial structured agents: presentation in the form of mash (MF) or grain (GF), the use of veterinary treatment or not (T vs. NT), and their interaction.

### 2.3. Statistical Analysis

The GENMOD procedure in the SAS Statistical Software Program (SAS Systems for Windows) was used with a multivariate linear model, and a binomial distribution was assumed. The standard errors were adjusted with a dispersion parameter that was estimated from the Pearson residuals. Corrections for the standard errors were included for the Pearson residuals. Analysis of variance (ANOVA) was implemented to study the significance of each effect using type III contrasts. Differences between groups were studied by a post hoc analysis, with 0.05 being considered the level of significance.

Adjusted by least squares means, each categorical variable was corrected using one of the following calculation procedures: (i) adjustment with respect to the general average of the rest of continuous variables and considering a uniform data distribution for the rest of the categorical variables, and (ii) the use of the “BYLEVEL” order of the SAS that allows the corrected averages for other variables to be corrected in the real conditions of production within a certain category for this database.

## 3. Results

**Figure 1:**
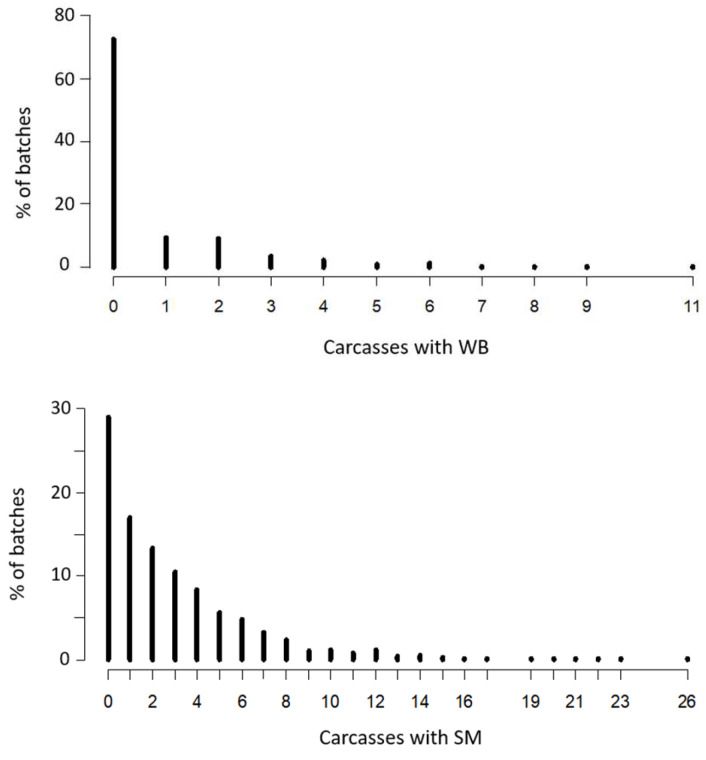
Incidence of distribution for wooden breast (WB) and spaghetti meat (SM).

The incidence of distribution for each myopathy is shown in Figure 1. Data did not conform to a normal model; thus, a binomial distribution was assumed in order to analyze the results. These results also showed that, of the 96 carcasses tested per batch, 75% showed no signs of WB. This percentage was only 30% in relation to the incidence of SM. The maximum number of carcasses affected by WB between all of the inspected batches was 11. This result was less than that observed for SM (26 carcasses).

Table 1 shows the average values of the production parameters and other continuous variables, such as WB and SM, expressed as percentages in order to characterize the broilers selected for the study. Our results showed a medium CV for mortality (5.46%), with a minimum value of 0.05% and a maximum of 9.96%, while the feed conversion ratio (FCR) showed a lower CV (3.91%), indicating good homogeneity between the batches in the study and an average value of 1.72 kg feed/kg live weight.

The frequency of muscle abnormalities was relatively low. The observed incidence of WB was 0.69% ± 1.47%, but there was a wide range of variation between sample units (0–11.5%). On the other hand, the incidence of SM had an average value of 2.94% ± 3.53% and a higher rate of 0–27.1%. A correlational analysis was carried out between the dependent variables without taking into account the effects of the broiler category or the form that the feed was presented in. Only weak correlations were detected. For example, weight had a direct positive correlation with the incidence of WB (*r* = 0.43) but a negative correlation with SM (*r* = −0.28).

The results of the analysis of variance for the effect of the variables that were determined to have a significant influence on the incidence of WB and SM are shown in Table 2. From these results, we can deduce that the average life weight has a significant effect (*p* < 0.001) on the incidence of WB and that it increases by a ratio of 1.11 (exp 1.07 × 0.1) for each 100 g increase in the slaughter weight. However, weight did not significantly (*p* > 0.05) affect the incidence of SM. On the other hand, an increase of 0.1 kg/kg in the FCR implied a decrease (*p* < 0.001) in the incidence of both breast defects (in a ratio of 0.594 (exp −5, 20 × 0.1) and 0.850 (exp −1.62 × 0.1), respectively). In contrast, the stocking density did not influence any of the breast-quality parameters analyzed in this study when the other covariates were present in the model. Broiler category had a significant effect on both myopathies (*p* < 0.001).

The effect of the animal category on the incidence of WB and SM is shown in Table 3. The results suggest that significant differences were dependent on broiler sex and breast abnormalities. WB incidence was higher for male than female broilers (*p* < 0.05), while differences due to color were not significant (*p* > 0.05). In all cases, the main myopathy was SM, the mean values of which varied between 2.77% and 5.34% for females.

The prevalence of WB and SM (expressed as a percentage) depending on the feed particle size and whether or not veterinary treatments were used is shown in Table 4. The supply of feed in the form of mash tended to significantly reduce the incidence of WB (*p* < 0.01), with no significant differences being observed between the groups in which veterinary treatments were used or not (*p* > 0.05). On the other hand, the incidence of SM showed the opposite effect, although to a lesser degree. The effects of medication were lower than those related to the form of the feed and did not present a clear trend.

## 4. Discussion

### 4.1. Production Parameters and Breast Myopathies

Vasdal et al. [31] indicated that flock weight uniformity can be expressed as the CV of the individual body weights. In our results, it can be seen that the CV of flock weight was over 10% (14.2%), which is associated with uniformity [32,33]. This result is in accordance to the data reported in broiler males (14.2%) by Griffin et al. [34] and similar to the 12.8% detected in broiler females. Gous [35] explained that the average faster growth rate of male broilers translates into poor uniformity in mixed-sex flocks. Thus, our results may be caused by differences between batches due to sex, as well as by the effect of several production measures. Vasdal et al. [31] reported that poor flock uniformity was associated with increased mortality and a reduced growth rate.

The incidence of WB observed in Table 1 is in accordance with reports by other authors, such as Huang and Ahn [36], who estimated that the incidence rates for this myopathy with severe conditions were present in approximately 5–10% of commercially produced breast fillets. Petracci et al. [17] detected the presence of SM in 20% of samples studied. In accordance with these results, Baldi et al. [37] reported an incidence of 21% in 16,000 breasts analyzed in Italy, while Montagna et al. [38] observed a 10% incidence of SM in 5580 broilers analyzed in Brazil. In agreement with our results, the CVs of both breast defects were high: 213% for WB and 120% for SM.

Some authors recognized that the prevalence of WB increases significantly with a higher growth rate, as well as a higher body weight [39,40], and this was also the case according to our results. However, there are not enough studies that confirm this trend for SM [41].

Although no significant differences were observed in the incidence of the myopathies due to the stocking density in this study, Feddes [32] reported that the stocking density had a significant effect on broiler performance and carcass traits. Zanetti et al. [42] also detected significant differences (*p* > 0.05) in terms of a smaller stocking density and the occurrence of WB.

### 4.2. Broiler Category and Feed Particle Form on the Prevalence of Breast Myopathies

A higher incidence of WB was also detected in male broilers than in females by other authors, such as Trocino et al. [7], who observed a WB prevalence of 16.3% in broiler males against an 8% prevalence in broiler females. However, SM is different [30,43], and mixed batches tend to produce intermediate results.

The skin color of a broiler can be changed by feed additives in order to satisfy consumer preferences. Nonetheless, skin color also depends on the abilities of the broiler to produce melanin pigment and to absorb and store carotenoids in the epidermis [44,45]. Bianchi et al. [46] reported a positive correlation between the yellowness of the skin and the yellowness of the breast meat. Different studies also analyzed how the color of poultry breast affects certain myopathies (e.g., [47]). However, there are no references to a relationship between skin color and the incidence of breast abnormalities.

The reduction in particle feed size may increase digestive efficiency and may consequently have a positive effect on carcass yield [27,48]. No previous studies were found to confirm that mash form feed, with or without veterinary treatments, reduces the incidence of WB. However, Joardar et al. [49] reported a lower incidence of WB in broilers whose diets were supplemented with fine limestone compared to chicks fed coarse limestone diets.

## 5. Conclusions

The present communication provides evidence about the incidence of wooden breast and spaghetti meat in real production conditions, which was lower than that reported by other authors from different countries. A possible linkage between these myopathies and live weight, the feed conversion ratio, and feed form was shown in this research and was determined to be dependent on sex and skin color. Further work should elucidate the common causative basis between the variables in the model and the incidence of WB and SM. It could be interesting to implement corrective measures in broiler production and in more studies in order to prevent the appearance of these muscular alterations.

## Figures and Tables

**Table 1:** Average values and variation in the continuous variables.

Productivity Parameters	Units	Average	Rate	CV ^1^
Density	kg/m^2^	36.5	14.5–49.8	17.1
Live weight	kg	2.72	1.44–3.77	14.2
Mortality	%	3.77	0.05–9.96	5.46
Feed conversion ratio	kg/kg	1.72	1.53–2.15	3.91
Growth rate	g/day	62.9	42.7–79.9	11.5
**Myopathies Incidence**	**Units**	**Average**	**Rate**	**CV**
Wooden breast	%	0.69	0.00–11.5	213
Spaghetti meat	%	2.94	0.00–27.1	120

^1^ CV: coefficient of variation (%).

**Table 2:** Significant effects of the variables studied on the incidence of wooden breast (WB) and spaghetti meat (SM).

Factor	Coefficient	Standard Error	Significance Level (*p*)
Wooden breast (WB)			
Intercept	−0.776	1.611	0.630
Broiler category			<0.001
YF	1.20	0.343	<0.001
YM	1.95	0.340	<0.001
YX	2.19	0.600	<0.001
WF	1.19	0.425	0.005
WM	1.96	0.304	<0.001
WX	1.61	0.379	<0.001
YF-65	0.644	0.455	0.157
WF-O	0.343		
Feed particle size			0.002
GF-T	−0.382	0.126	0.003
GF-NT	−0.260	0.129	0.045
MF-T	−0.936	0.321	0.004
MF-NT	−0.640	0.255	0.012
Live weight (kg)	1.07	0.224	<0.001
FCR (kg/kg)	−5.20	0.881	<0.001
Spaghetti meat (SM)			
Intercept	−0.363	0.797	0.648
Broiler category			<0.001
YF	0.27	0.084	0.01
YM	−1.54	0.152	<0.001
YX	−0.694	0.471	0.14
WF	0.01	0.113	0.38
WM	−1.23	0.082	<0.001
WX	0.132	0.138	0.34
YF-65	−0.241	0.109	0.027
WF-O	0		
Feed particle size			0.002
GF-T	−0.209	0.077	0.006
GF-NT	−0.144	0.083	0.084
MF-T	−0.433	0.122	<0.001
MF-NT	−0.219	0.106	0.038
FCR (kg/kg)	−1.62	0.456	<0.001

Broiler categories: yellow female (YF), yellow male (YM), yellow mixed (YX), white female (WF), white males (WM), white mixed (WX), yellow female with a 65% cereal on diet (YF-65), white female in over-density (WF-O). Feed particle size: grain feed with medication treatment (GF-T), grain diet without medication treatment (GF-NT), mash diet with medication treatment (MF-T), mash diet without medication treatment (MF-NT). FCR: feed conversion ratio.

**Table 3:** Incidence of WB and SM (expressed as a percentage) for each category of broiler (means adjusted by least squares in the natural transformed scale).

Broiler Category	WB	SEM	SM	SEM
YF	0.250 ^bc^	0.054	5.340 ^a^	0.304
YM	1.060 ^a^	0.115	0.979 ^b^	0.136
YX	0.511 ^abc^	0.273	2.230 ^ab^	1.020
WF	0.206 ^bc^	0.067	4.030 ^a^	0.368
WM	0.891 ^a^	0.060	1.400 ^b^	0.084
WX	0.575 ^ab^	0.145	5.670 ^a^	0.66
YF-65	0.084 ^c^	0.030	2.770 ^a^	0.218
WF-O	0.091 ^c^	0.024	4.740 ^a^	0.208

^a–c^ Means within the same column that have no common letters that differ significantly (*p* < 0.05). Broiler categories: yellow female (YF), yellow males (YM), yellow mixed (YX), white female (WF), white Males (WM), white Mixed (WX), yellow female with a 65% cereal on diet (YF-65), white female in over-density (WF-O). WB: wooden breast. SM: spaghetti meat. SEM: standard error of the mean.

**Table 4:** Incidence of WB and SM (expressed as a percentage) depending on the feed form presentation and the use or not of veterinary treatments.

Feed Particle Size	WB	SEM	SM	SEM
GF-T	0.493 ^a^	0.047	2.080 ^a^	0.118
GF-NT	0.406 ^a^	0.039	2.300 ^a^	0.110
MF-T	0.112 ^b^	0.029	2.560 ^a^	0.210
MF-NT	0.116 ^b^	0.037	2.290 ^a^	0.235

^a, b^ Means within the same column that have no common letters differ significantly (*p* < 0.05). Feed particle size: grain feed with medication treatment (GF-T), gain diet without medication treatment (GF-NT), mash diet with medication treatment (MF-T), mash diet without medication treatment (MF-NT). WB: wooden breast. SM: spaghetti meat. SEM: standard error of the mean.

## Data Availability

Not applicable.
